# Effect of Dietary Gluten on Dendritic Cells and Innate Immune Subsets in BALB/c and NOD Mice

**DOI:** 10.1371/journal.pone.0118618

**Published:** 2015-03-04

**Authors:** Jesper Larsen, Christian Weile, Julie Christine Antvorskov, Kåre Engkilde, Signe Marie Borch Nielsen, Knud Josefsen, Karsten Buschard

**Affiliations:** The Bartholin Institute, Rigshospitalet, 2100, Copenhagen, Denmark; La Jolla Institute for Allergy and Immunology, UNITED STATES

## Abstract

The innate immune system is known to play an important role in oral tolerance to dietary antigens. This is important in development of celiac disease (CD) but may also be important in type 1 diabetes (T1D), and could potentially explain the reduced incidence of T1D in mice receiving a gluten-free (GF) diet. The direct *in vivo* effect of gluten on innate cells, and particularly dendritic cells (DC) is not sufficiently clarified. Therefore, we wished to investigate the innate cell populations of spontaneous diabetic NOD mice and healthy BALB/c mice kept on a GF or a standard (STD) gluten containing diet. We studied, by flow cytometry and reverse transcription-quantitative polymerase chain reaction (qRT-PCR), if dietary gluten induces changes in the activation of DCs and distribution of selected innate cells in lymphoid, pancreatic and intestinal tissues in BALB/c and NOD mice. We found that a GF diet increased the percentage of macrophages in BALB/c spleen and of CD11c^+^ DCs in BALB/c and NOD spleen. Strictly gluten-free (SGF) diet increased the percentage of CD103^+^ DCs in BALB/c mice and decreased percentages of CD11b^+^ DCs in mesenteric and pancreatic lymph nodes in BALB/c mice. SGF diet in BALB/c mice also decreased DC expression of CD40, CCR7 and MHC-II in pancreatic lymph nodes. In conclusion, GF diet changes the composition of the innate immune system in BALB/c and NOD mice and increases expression of DC activation markers in NOD mice. These results contribute to the explanation of the low diabetes incidence in GF NOD mice. This mechanism may be important in development of type 1 diabetes, celiac disease and non-celiac gluten sensitivity.

## Introduction

Oral tolerance to dietary antigens is important in healthy individuals and relies on the innate immune system and presentation by dendritic cells (DCs) [1]. In celiac disease (CD), intolerance to indigestible wheat gluten proteins results in chronic intestinal inflammation [2]. The initiation of T1D has also been linked to intake of gluten, and an increasing number of publications describe a gluten related disorder coined non-celiac gluten sensitivity [3,4]. A gluten-free (GF) diet has been shown to prevent diabetes in NOD mice [5] and Biobreeding (BB) rats [6]. T1D patients without CD have an abnormal gluten response [7,8], and a gluten-free diet is reported to halt disease progression in a case study [9]. Both T1D and CD share high susceptibility associated with the HLA-DQ2 and HLA-DQ8 haplotypes [10,11].

The development of CD and T1D involves cell types from both the innate immune and adaptive immune system [12,13]. Neutrophils, pDCs and B-cells infiltrate the pancreas of NOD mice as early as 2 weeks after birth, and several innate cell types are required for the autoimmune response [14].

We have shown that cell populations of the adaptive immune system such as regulatory T cell subsets, T helper type 17 (Th17) and CD8^+^ cells are affected by gluten exposure in NOD mice and BALB/c mice [15–17]. Gliadin fragments are also known to stimulate several different innate cell population such as macrophages, eosinophils, mastcells and monocytic cells in vitro [18–21], and we have recently shown that gliadin increases NK cell cytotoxicity, cytokine production and expression of NKG2D ligands in intestinal tissue and pancreatic islets [17,22]. Another study shows that stimulation with α-amylase/trypsin (ATI) inhibitors from the gliadin fraction result in a strong innate response via TLR-4 involving DCs, macrophages and monocytes in intestinal inflammation in vivo and in vitro [23].

Gliadin stimulation in vitro activates bone marrow-derived DCs from BALB/c mice [24] and human monocyte-derived DCs [19,25]. Further, gliadin peptides increases the crosstalk between NK cells and DCs [26]. Only few studies have been performed to clarify the mechanisms by which gluten affect dendritic cells in vivo. One of them shows that DCs in celiac lesions from human patients, display a unique activated phenotype which activates gluten-reactive T cells in vitro [27] and another shows that GF celiac patients accumulate CD14^+^CD11c^+^ DCs in gut mucosa after in vivo gluten challenge [28].

It is well known that dietary gluten increases intestinal permeability in CD, and increased permeability is also seen in T1D patients and animal models of the disease [29–31]. The intestinal immune system has proven important for development of T1D, as lymphocytes that have a mucosal phenotype and have been primed in the gut, accumulate in the prediabetic pancreas in NOD mice [32,33].

The aim of the present study was to investigate the effect of dietary gluten on intestinal and lymphoid DC subsets and other innate cell populations in BALB/c and NOD mice. The innate immune system has emerged as an important component of the pathogenesis of T1D [34], and may also play a role in non-celiac gluten sensitivity. Therefore we studied the effects of a GF diet on DCs, plasmacytoid DCs, neutrophils, B-cells and macrophages in lymphoid organs from BALB/c and NOD mice.

DCs are able to activate both the innate and adaptive immune system, and are responsible for regulating oral tolerance and immunity in the intestine [35,36]. Further gliadin is known to stimulate several innate parameters *in vitro* using cells from non-diabetic BALB/c mice. Therefore we looked specifically at *in vivo* DC activation markers and subpopulations in pancreatic and intestinal lymph nodes in BALB/c mice receiving either GF or STD diet.

## Materials and Methods

### Ethics

Animal experiments were conducted at the University of Copenhagen in accordance with the revised Council of Europe Convention ETS 123, the revised EU directive 2010/63 and the Danish Proclamation of law on animal experimentation 1306 from 23.11.2007, and was approved specifically by the The Animal Experiments Inspectorate (AEI), Danish Veterinary and Food Administration (license no. 2012-15-2934-00086). The protocol was approved by the Department of Experimental Medicine, Faculty of Health and Medical Sciences, University of Copenhagen, Denmark (project no. P 14–137).

### Animals and diet

BALB/CJBomTac were purchased from Taconic Europe A/S, Ry, Denmark and NOD mice were from Taconic US. The animals received standard (STD), non-purified Altromin diet, or a GF, modified Altromin diet (Altromin, Lage, Germany) from 4 weeks of age. These two diets have previously been used at The Bartholin Institute to study the effect of a GF diet on diabetes incidence in NOD mice. The exact composition of the STD and the GF diet is given in [37]. To study the effect of timing of gluten exposure, we bought breeding pairs of BALB/c and NOD mice (Taconic US, Taconic Europe A/S, Ry, Denmark) and exposed them to either GF or STD diet during breeding, exposing the puppies to only one diet both in uterus, during weaning and after weaning. These groups of animals were named strictly gluten-free (SGF) and standard-diet (STD).

The mice were kept in a specific pathogen-free (SPF) animal facility (temperature 22±2 degrees, 12h light cycle, air changed 16 times per hour, humidity 55±10%) with free access to water and food.

Flow cytometric studies for description of innate cell subsets were done on 13-week-old female BALB/c and NOD mice on a GF vs a STD diet (9 mice in each group). Flow cytometric studies for description of DC markers were done on 9-week-old female BALB/c mice on a SGF vs a STD diet (20 mice in each group).

Female SGF and STD mice (12 in each group) were used in the study at 8, 13 and 20 weeks of age to study if the effect of diet was in the prediabetic fase or later in disease development. Tissues used for RT-qPCR were also used in [17] and [22].

### Cell purification and flow cytometry

Mice were sacrificed and spleen (S), auricular lymph nodes (ALN), pancreas draining lymph nodes (PLN), mesenteric lymph nodes (MLN), Peyer’s patches (PP) and liver (L) were isolated from 9- or 13-week-old BALB/C or NOD mice on the respective diets. Organs were mechanically homogenized and passed through a 70 μm cell strainer to obtain single cell suspensions. Cells from 2–3 mice were pooled for each organ, and single-cell suspensions were prepared. Dead cell discrimination was performed before fixation, using AmCyan conjugated LIVE/DEAD fixable aqua dead cell staining kit (L34957, Invitrogen) to exclude dead cells. Cells were incubated with Fc block (CD16/CD32) for 10 minutes before staining with mAb to reduce Fc receptor-mediated binding (553141, BD). Subsequently cells were surface-stained using mAb (for complete list see supplementary information) for 30 minutes before fixation in 2% PFA. For compensation controls we used ArC Amine Reactive (for LIVE/DEAD stain) and AbC anti-Rat/Hamster Bead Kits (Invitrogen). The cells were analysed by flow cytometry using a LSR-II (BD Bioscience), and data were analysed with use of Flowlogic (Inivai Technologies) software. For gating, we used fluorescence minus one (FMO) controls. Complete list of antibodies (S1 Text).

### Quantitative RT-PCR (qRT-PCR)

RNA was extracted from a number of tissues for qPCR analysis as described in detail previously, in a previous study were the same RNA material is used to study the expression of NKG2D and NKG2D ligands [17]. In short, duodenum and islets were isolated from GF and STD BALB/c and NOD mice at 13 weeks of age. Duodenum sections, whole pancreas, spleen and PLN were isolated at 8, 13 and 20 weeks of age, from NOD mice on SGF and STD diet. Islets were isolated from 13 weeks old mice on SGF and STD diet.

Specific mRNA levels were quantified on a Lightcycler II (Roche, Penzberg, Germany) using SYBR II qPCR mixture (Takara Bio, Otsu, Japan), and normalized to the housekeeping gene beta actin as previously described [17]. Primers (see supplemental materials) were designed using Primer3 software [38] and synthesized by TAG Copenhagen (Copenhagen, Denmark). Complete list of primers (S2 Text)

## Results

### GF diet increases proportions of macrophages in BALB/c spleen and dendritic cells in BALB/c and NOD spleen

Using flow cytometry, we examined the composition of different innate populations in spleen from 13-week-old BALB/c and NOD mice receiving STD gluten-containing diet and GF diet from the age of 4 weeks.

**Fig 1:**
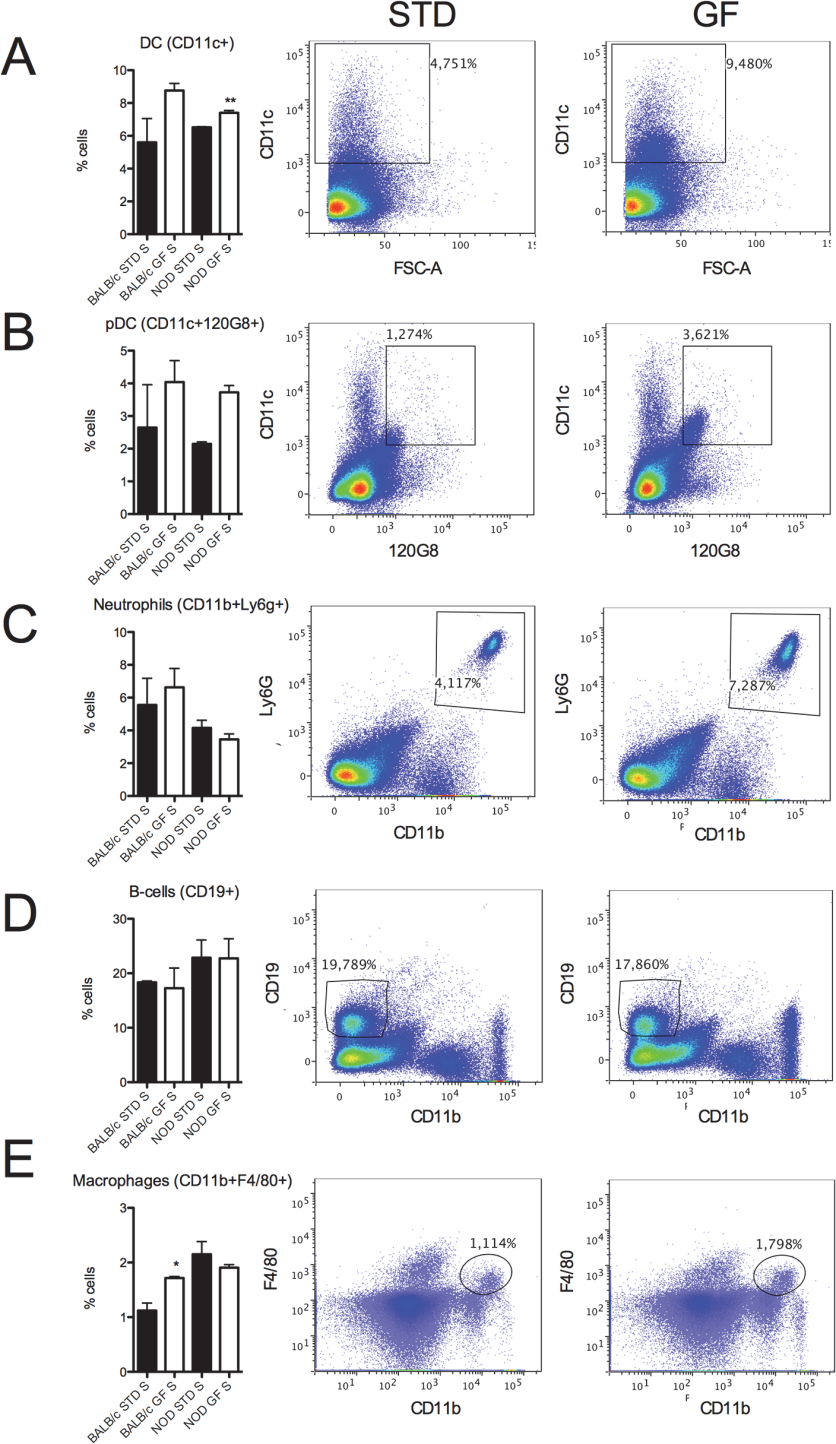
Profile of innate cell populations (FACS). Flow cytometric analysis of cells isolated from spleen (S) from BALB/c and NOD mice receiving STD or GF diet. Bars (left) represent percentages of cells and FACS plots (right) show representative stainings of BALB/c spleen cells from STD and GF mice. (A) Dendritic cells (CD11c^+^), (B) Plasmacytoid dendritic cells (CD11c^+^120G8^+^), (C) Neutrophils (CD11b^+^Ly6G^+^), (D) B-cells (CD19) and (E) Macrophages (CD11b^+^F4/80^+^). Data are represented as mean values ± standard error of the mean (n = 3). * p < 0.05; ** p < 0.01.

In NOD mice we found a 13.5% increase in CD11c^+^ cells, a marker for DCs, in animals receiving the GF diet compared to animals on a STD diet (p = 0.035, Fig. 1A). No significant changes were observed in B cells (CD19^+^ cells) or neutrophils (CD11b^+^Ly6G^+^ cells) (Fig. 1C and 1D). Finally, we found a 53% increase in CD11b^+^F4/F80^+^ macrophages in BALB/c spleen in mice receiving the GF diet (p = 0.0139, Fig. 1E). Increase in DCs in BALB/c mice and in pDCs in BALB/c and NOD spleens of mice receiving the GF diet, were not significant (Fig. 1B).

### qPCR innate populations

**Fig 2:**
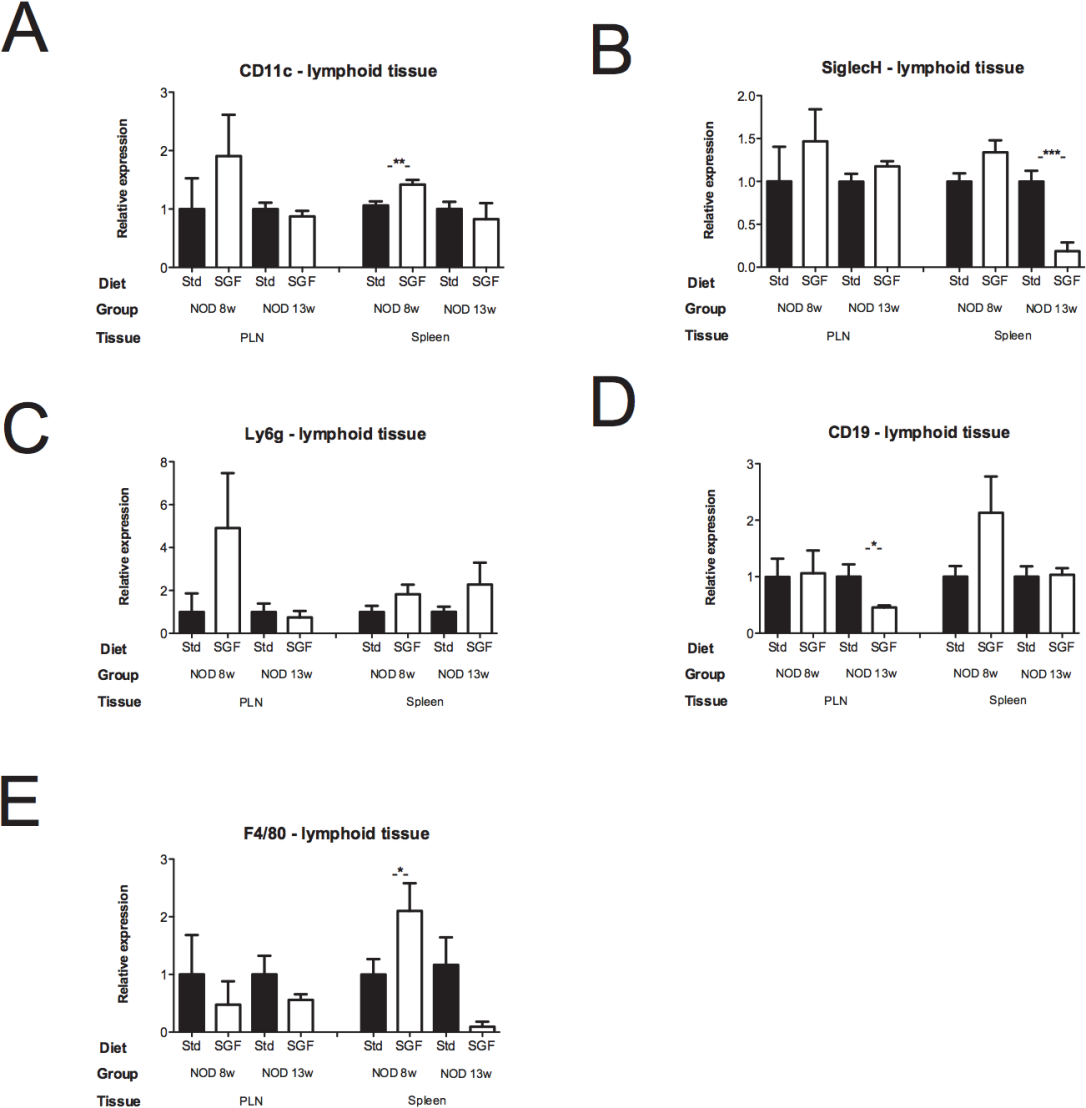
mRNA expression of innate cell markers. Relative mRNA expression levels of (a) CD11c, (b) SiglecH, (c) Ly6G, (d) CD19 and (e) F4/80 in PLN and spleen from 8 and 13-week old mice kept on a gluten- strictly gluten-free (SGF) or matched control standard diet (STD). Right panel: Expression levels in PLN and Spleen from SGF and STD NOD mice at 8 and 13 weeks. Data are represented as mean values ± standard error of the mean (n = 3). * p < 0.05; ** p < 0.01; *** < 0.001.

We further analysed the mRNA expression of innate cell markers in intestine, pancreas and lymphoid organs from NOD and BALB/c mice. CD11c expression was increased by 34% in spleens from 8-week-old mice that received SGF (p = 0.0041), matching the data for CD11c in Fig. 1. Expression of CD11c was increased by 34% (p<0.01, Fig. 2A) in S from SGF NOD mice at 8 weeks of age. Expression of SiglecH (pDC marker), was reduced by 81% in S from SGF NOD mice at 13 weeks (p<0.001, Fig. 2B). CD19 (B cell marker) expression was reduced with 54% in PLN from SGF mice at 13 weeks (p = 0.03, Fig. 2D), and F4/80 (macrophage marker) was 2-fold increased in spleen from SGF mice at 8 weeks (p<0.05, Fig. 2E).

In the pancreas, we observed a pattern of innate mRNA upregulation in SGF NOD mice. Expression of innate cell markers was significantly higher in SGF NOD pancreas at 8, 13 or 20 weeks of age. Only small changes were seen in isolated islets from NOD mice (S1 Fig.).

In intestinal tissue we generally find reduced mRNA expression of innate cell markers in SGF NOD mice. Most significant changes are seen at 13 weeks of age.

### Surface expression of CD11c^+^ DC surface markers in SGF BALB/c mice

**Fig 3:**
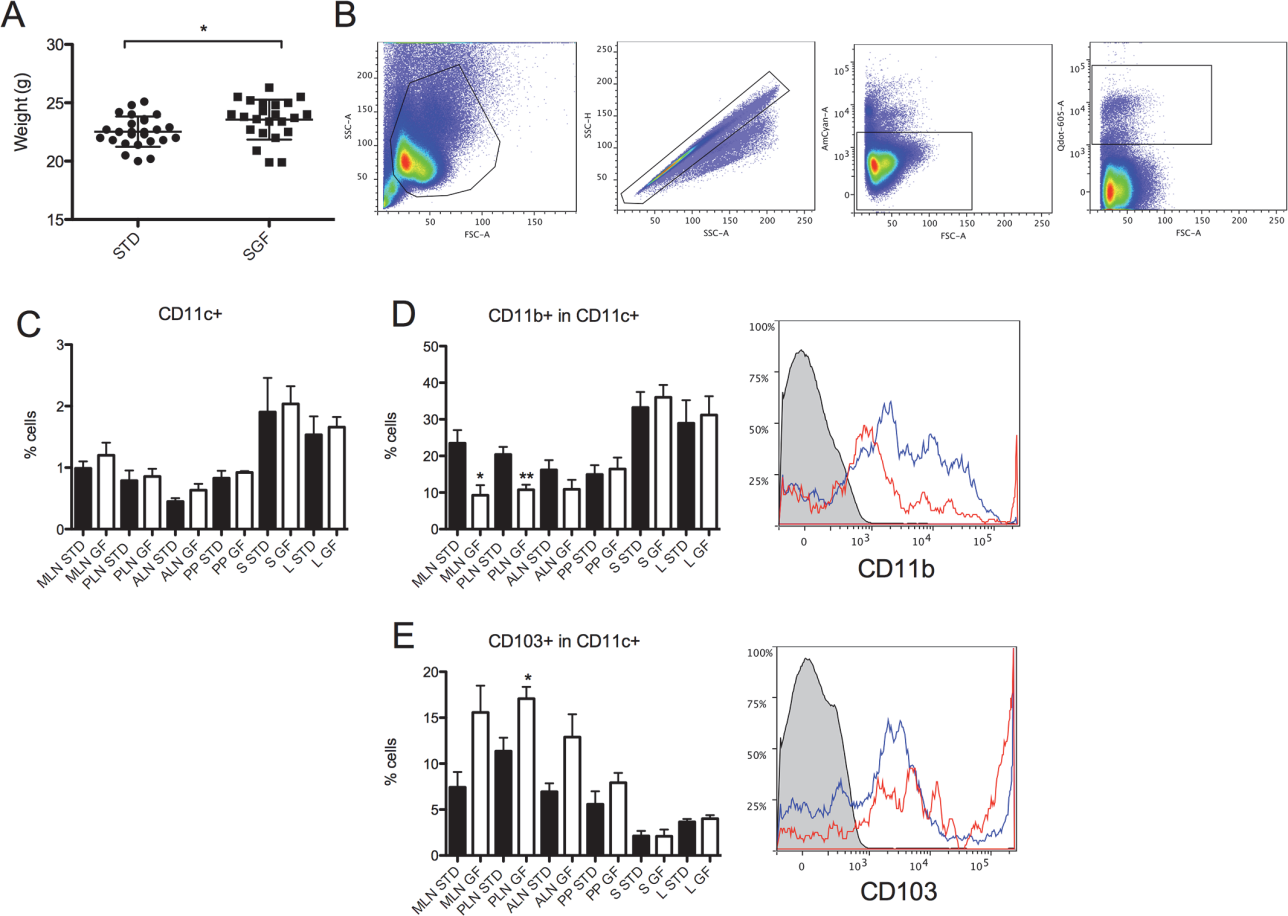
Reduced levels of CD11b^+^ DCs and increased levels of CD103^+^ DCs in MLN and PLN of SGF BALB/c mice. Flow cytometric analysis of cells isolated from mesenteric lymph nodes (MLN), pancreatic lymph nodes (PLN), auricular lymph nodes (ALN), Peyer’s patches (PP), spleen (S) and liver (L) from BALB/c mice. (A) Weight data for BALB/c mice used in the flow cytometry (n = 23–26). (B) Representative FACS plot showing lymphocyte, singlet, live and CD11c^+^ gating in BALB/c SGF spleen. (C) Bars represent percentages of CD11c^+^ dendritic cells in BALB/c mice in the STD (black bars) or SGF group (white bars). (D) Bars represent percentages of CD11b^+^CD11c^+^ dendritic cells after gating on CD11c^+^ cells. Histogram represent the expression of CD11b^+^ after gating on CD11c^+^ cells in unstained control (grey), STD (blue line), SGF (red line). (E) Bars represent percentages of CD103^+^CD11c^+^ dendritic cells after gating on CD11c^+^ cells. Histogram represent the expression of CD103 after gating on CD11c^+^ cells in unstained control (grey), STD (blue line), SGF (red line). Data are represented as mean values ± standard error of the mean (n = 3–5). * p < 0.05; ** p < 0.01.

As we observed the most significant diet-induced changes in CD11c^+^ cells, we further investigated the specific changes seen in CD11c^+^ dendritic cells in BALB/c mice receiving a STD vs GF diet, but now also including the diet fed to the mothers during pregnancy. We observed a small but significant weight increase of 1g in the mice receiving SGF diet, compared to STD diet (p = 0.021, Fig. 3A). We included several organs (MLN, PLN, ALN, PP, S and L) from 9-week-old BALB/c and analysed expression of CD11c, CD103, CD11b, and DC activation markers using flow cytometry. We observed a small increase in percentage of CD11c^+^ DCs in all organs of BALB/c mice receiving the SGF diet, although not any significant changes, and a smaller increase than the one observed in GF animals (Fig. 3C).

### SGF diet increases CD103^+^ DCs, but decrease CD11b in BALB/c PLN

CD11b^+^ DCs has been shown to increase in numbers in murine insulitis, but has also been shown to be tolerogenic and suppress disease in the NOD mouse [39,40].

CD11b^+^ cells were significantly decreased in the CD11c^+^ population by 60.4% in MLN and 47.1% in PLN from SGF BALB/c mice (p = 0.02 and p = 0.0084 respectively, Fig. 3D). Changes in other organs were non-significant.

The proportion of CD103^+^ cells in the CD11c^+^ population was increased by 50.4% in BALB/c PLN in mice receiving SGF diet (p = 0.026, Fig. 3E). The same pattern emerged in MLN, ALN and PP without being significant.

### SGF diet decreases DC activation markers MHC-II, CD40 and CCR7 on CD11c^+^ cells in BALB/c PLN

DCs can effectively process and present peptides to T-cells and the outcome may be tolerogenic or inflammatory, depending on the state of maturation, pro-inflammatory signals and their environment [41].

To characterize the phenotypic changes on DCs induced by gluten, we included several DC surface activation markers in the flow cytometric analysis.

**Fig 4:**
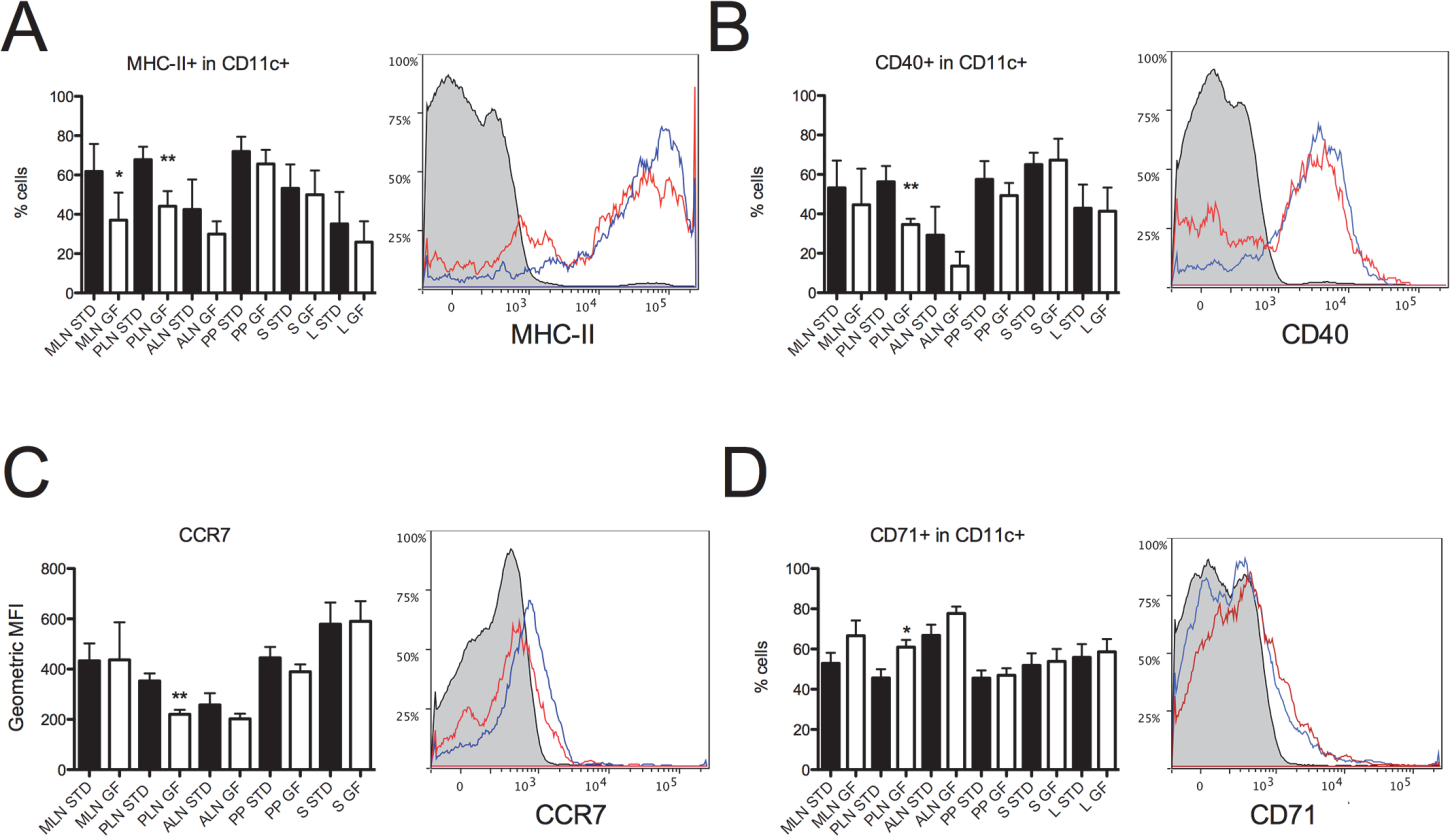
Reduced levels of MHC-II^+^, CD40^+^ and increased levels of CD71^+^ DCs in SGF BALB/c PLN. Flow cytometric analysis of cells isolated from mesenteric lymph nodes (MLN), pancreatic lymph nodes (PLN), auricular lymph nodes (ALN), Peyer’s patches (PP), spleen (S) and liver (L) from BALB/c mice receiving STD or SGF diet. (A) Bars represent percentages of MHC-II^+^CD11c^+^ dendritic cells after gating on CD11c^+^ cells. (B) Bars represent percentages of CD40^+^CD11c^+^ dendritic cells after gating on CD11c^+^ cells. (C) Bars represent percentages of CD71^+^CD11c^+^ dendritic cells after gating on CD11c^+^ cells. Data are represented as mean values ± standard error of the mean (n = 3–6). * p < 0.05; ** p < 0.01.

Gating on CD11c positive cells we found differences in the expression of the DC activation surface markers MHC-II, CD40 and CCR7. Some of the biggest changes were seen in the PLN. For MHC-II we found a 40% reduction in the percentage of MHC-II^+^ cells in the CD11c^+^ population in SGF MLN and a 35% reduction in SGF PLN (p = 0.0463 and p = 0.0034 respectively, Fig. 4A). CD40 is decreased by 38% in SGF PLN (p = 0.002, Fig. 4B). The geometric mean fluorescence intenstity (MFI) of the chemokine receptor CCR7 is 37.6% lower in SGF PLN compared to STD PLN (p = 0.0091, Fig. 4D).

**Fig 5:**
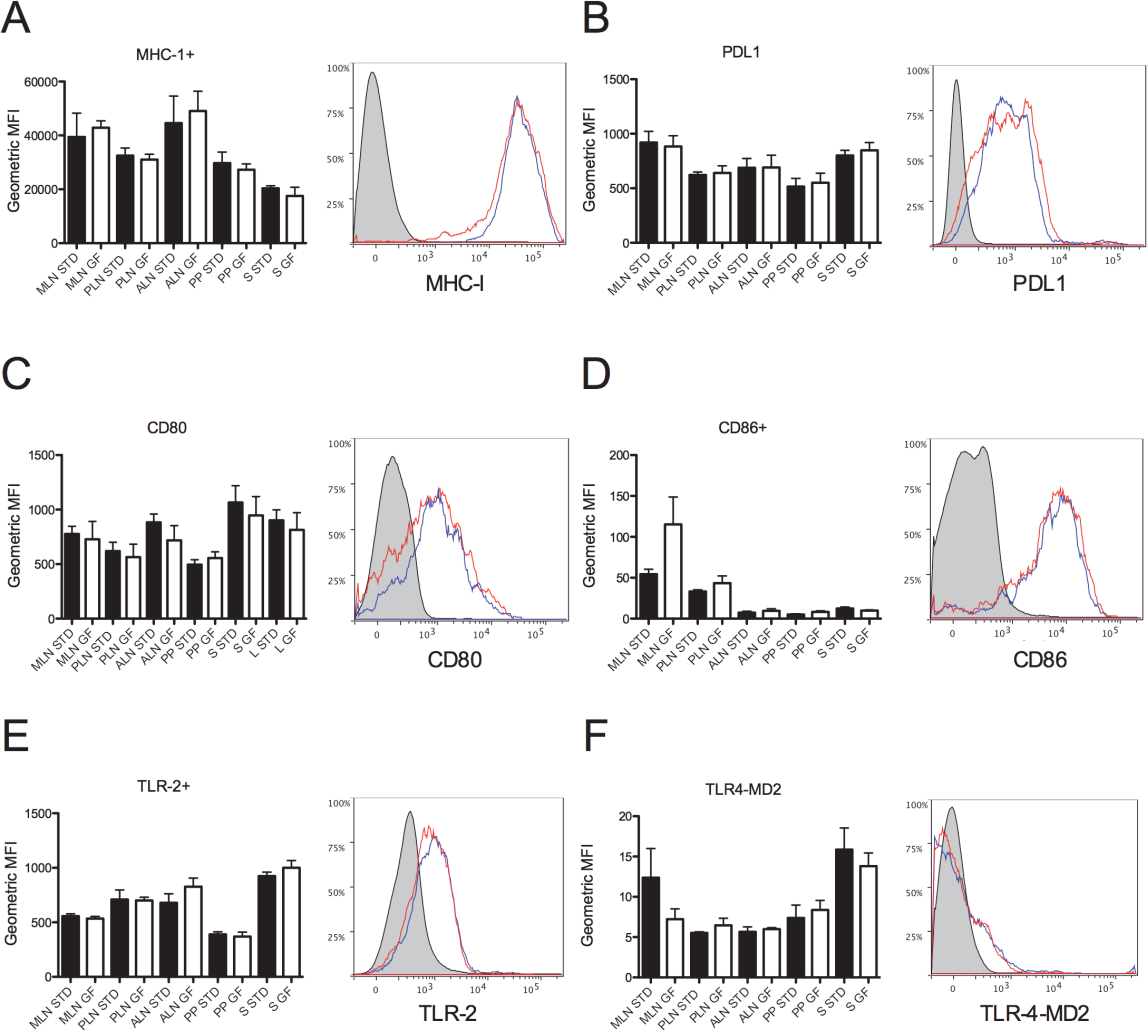
Dendritic cell surface expression. Expression of (a) MHC-1, (b) PDL1, (c) CD80, (d) CD86, (e) TLR-2, and (f) TLR-4-MD2, expressed as geometric MFI. Data are represented as mean values ± standard error of the mean (n = 3–6).

The general cellular activation marker CD71 is upregulated by 33.6% in SGF PLN (p = 0.0337). No difference was observed in the expression of MHC-1, PDL1, CD80, CD86, TLR-2 or TLR4 (Fig. 5A-F).

## Discussion

In this paper we have shown that dietary gluten affects a wide range of innate immune parameters in the lymphoid system, pancreas and intestine. GF diet increases the proportions of CD11c^+^ DCs in NOD spleen and CD11b^+^F4/80+ macrophages in BALB/c spleen. Further GF and SGF diet increase the mRNA expression of CD11c, SiglecH, Ly6G, CD19 and F4/80 in pancreatic tissue, but substantially reduce the expression in intestinal tissue from NOD mice.

We did not see any diet-induced changes in the percentage of CD11c^+^ cells in SGF BALB/c mice, but we found a decrease in CD11b^+^CD11c^+^ DCs in the MLN and PLN in mice on a SGF diet. Similar results have been described in offspring of GF NOD mice [42], and thereby seem to be a general effect of gluten.

Conventional DCs in islets can be divided in two functionally distinct main subsets, consisting of CD103^+^ DCs and CD11b^+^ DCs. Interestingly the percentage of CD11b^+^ DCs increases during insulitis, and the inflammation induces phenotypic changes. The CD103^+^ phenotype is unaffected by islet inflammation, and the cells are less effective than CD11b^+^ DCs in uptake of soluble antigens [39].

Interestingly, we also found increased percentages of CD103^+^CD11c^+^ DCs in MLN and PLN in BALB/c mice fed a SGF diet. It has been reported that the density of CD103^+^ DCs is decreased in the celiac lesion in celiac patients [43] and the cells play an important role in tolerance of food antigens in the intestinal mucosa [44]. CD103^+^ DCs migrate to gut draining lymph nodes where they initiate adaptive immune responses [45]. Tolerogenic CD103^+^ DCs in MLN have been shown to induce FoxP3^+^ Tregs [46] and the same might be the case in PLN. Interestingly, it has also been shown that CD103^+^CD11c^+^ DCs have an impaired ability to induce FoxP3^+^ Treg differentiation in T1D patients [47].

The increase expression in maturation and activation markers MHC-II, CD40 and CCR7 in BALB/c PLN and MLN in our study, is comparable to previously reported results in human monocyte-derived DCs from healthy donors [19] and BALB/c bone marrow derived DCs [24]. The activated dendritic cells may both activate innate immune cells and T cells. However we cannot conclude whether the activated DCs in PLN and MLN are tolerogenic or inflammatory. In a previous in vitro study in BALB/c mice, the gluten-activated DCs could not be classified as proinflammatory nor anti-inflammatory. Instead they secreted the murine IL-8 homogolous MIP-2 and keratinocyte-derived cytokine, which attracts neutrophils [24]. Further studies are necessary to dissect the effect of gluten on the T cell-stimulating potential of DCs from different lymphoid compartments. Further it would be interesting to look into the NK stimulating potential of gluten stimulated DCs in vivo, as in vitro studies with celiac patients show increased NK/DC crosstalking with gliadin stimulation, and we have shown effects of gluten on murine NK cells [22,26].

The most significant changes in DC activation markers following a SGF diet were seen exclusively in the MLN and PLN. This is not surprising, since cells in MLN are in direct contact with the intestinal gluten. Cells derived from MLN in BB rats are more proinflammatory in animals fed a wheat based diet [48]. Interestingly we find equally large changes in PLN, which could be explained by the shared lymphocyte circulation between gut-associated lymphoid tissue (GALT) and PLN [41].

Dietary gluten may affect innate cells in different locations in vivo. Gliadin is taken up by DCs in the lamina propria after disassembly of intestinal tight junctions and increased permeability [13], and it has also been suggested that intestinal DCs may sample gliadin peptides directly from the lumen [35]. After gliadin-loading in the intestine, DCs may migrate to pancreatic lymph nodes and activate autoreactive T cells [13]. The described changes seen in healthy BALB/c mice may be relevant in non-celiac gluten sensitivity, which is thought to be a disorder involving innate activation by gluten peptides [3,4].

The diabetogenic potential of gluten is affected by timing of introduction [49], which explains observed differences between GF and SGF animals. This demonstrates how exposure to gluten in utero and early postnatal may alter the immune system. The timing of gluten exposure is also important regarding the risk of islet autoimmunity in animal models [6,50] and humans, where gluten introduction between 3 and 7 months after birth, decreases risk of autoantibodies [51,52].

Collectively our data presented in this study, provide new insight into the innate stimulatory effect of dietary gluten *in vivo*. We find activation of innate cells populations in gut and pancreas from diabetes prone NOD mice but interestingly also in MLN and PLN from healthy BALB/c mice. The described effect of gluten on intestinal innate immune activation is known to be important in CD, but may also be important in non-celiac gluten sensitivity [4] and possibly development of T1D.

## Supporting Information

S1 Text Complete list of antibodies used in flow cytometric analysis. (DOCX)

S2 Text Complete list of primers used in RT-PCR analysis. (DOCX)

S1 Fig Pancreas and intestinal mRNA expression of innate cell markers. Relative mRNA expression levels of (a) CD11c, (b) SiglecH, (c) Ly6G, (d) CD19 and (e) F4/80 from 8 and 13-week old mice kept on a gluten-free (GF), strictly gluten-free (SGF), or matched control standard diet (STD) diet. Left panel: Expression levels isolated islets from SGF and STD NOD mice at 13 weeks, and expression in pancreas from SGF and STD NOD mice at 8, 13 and 20 weeks. Right panel: Expression levels in intestinal tissue from GF and STD BALB/c and NOD mice at 13 weeks, and SGF and STD NOD mice at 8, 13 and 20 weeks. Data are represented as mean values ± standard error of the mean (n = 6–12). * p < 0.05; ** p < 0.01; *** < 0.001.
(TIFF)

S2 Fig FACS plots. FACS plots showing representative plots of CD11b, CD40 and MHC-II in SGF and STD BALB/c PLN.
(TIFF)
