# Supplementary material for: Effect of Dietary Gluten on Dendritic Cells and Innate Immune Subsets in BALB/c and NOD Mice
Source: PLoS One. 2015 Mar 4;10(3):e0118618. doi: 10.1371/journal.pone.0118618 (PMC4349814; doi:10.1371/journal.pone.0118618)
Supplement: S1 Text — (DOCX) [file pone.0118618.s001.docx]

**S1 text. List of FACS antibodies**

The following monoclonal-antibodies (mAb) were purchased from BD Pharmingen: PerCP-Cy5.5-conjugated rat-anti mouse CD71 (562858), PE-conjugated rat-anti mouse CD103 (561043), PE-Cy7-conjugated rat-anti mouse CD11b (552850), BV605-conjugated hamster-anti mouse CD11c (563057), APC-conjugated rat-anti mouse CD40 (558695), FITC-conjugated hamster-anti mouse CD80 (553768), BV421-conjugated rat-anti mouse CCR7 (562675), APC-conjugated rat-anti mouse MHC-1 (558695), BV421-conjugated rat-anti mouse CD86 (562675), APC-H7-conjugated rat-anti mouse CD19 (560143)

The following monoclonal-antibodies (mAb) were purchased from eBioscience: APC-eFlour780-conjugated rat-anti mouse MHC-II (47-5321-82), APC-conjugated hamster-anti mouse CD11c (17-0114-82), eFlour450-conjugated rat-anti mouse F4/F80 (48-4801-82), eFluor660-conjugated rat-anti mouse Ly-6G (50-5931-82), PerCP-Cy5.5-conjugated rat-anti mouse CD11b (45-0112-82), FITC-conjugated rat-anti mouse TLR-2 (11-9021-82), PE-Cy7-conjugated rat-anti mouse TLR-4-MD2 (25-9924-82)

The following monoclonal-antibodies (mAb) were purchased from Imegenex: FITC-conjugated rat-anti mouse pDC (120G8.04) (DDX0390FITC)
