# Supplementary material for: Effect of Dietary Gluten on Dendritic Cells and Innate Immune Subsets in BALB/c and NOD Mice
Source: PLoS One. 2015 Mar 4;10(3):e0118618. doi: 10.1371/journal.pone.0118618 (PMC4349814; doi:10.1371/journal.pone.0118618)
Supplement: S2 Text — (DOCX) [file pone.0118618.s002.docx]

**S2 text. List of primers**

The following primers were used:

Ly6G:

Left primer: 5´-CCA TCT GCC CCA CTA CTC TG- 3´

Right primer: 5´-AAC CAG GCT GAA CAG AAG CA- 3´

CD11C (ITGAX):

Left primer: 5´-AGC AGG AGT GTC CAA AGC AA- 3´

Right primer: 5´-AAA GGG CTT GAC GTG GAG AT- 3´

SiglecH

Left primer: 5´-TCA GCA TCC CAC AGA CAC TG- 3´

Right primer: 5´-TGA CAG GTG AGG TTG GTT CC– 3´

F4/80 (EMR1)

Left primer: 5´-TCA TCA GCC ATG TGG GTA CA– 3´

Right primer: 5´-AAG AAG CAG GCG AGG AAA AG– 3´

CD19

Left primer: 5´-AAC CAC GTG ACT CCC AAG TG– 3´

Right primer: 5´-GGG TTT ATT TGG GGA AAG CA– 3´

Beta actin

Left primer: 5´-CAT TGC TGA CAG GAT GCA GA– 3´

Right primer: 5´-TTG CTG ATC CAC ATC TGC TG– 3´
