# Supplementary figures and images for: Effect of Dietary Gluten on Dendritic Cells and Innate Immune Subsets in BALB/c and NOD Mice
Source: PLoS One. 2015 Mar 4;10(3):e0118618. doi: 10.1371/journal.pone.0118618 (PMC4349814; doi:10.1371/journal.pone.0118618)

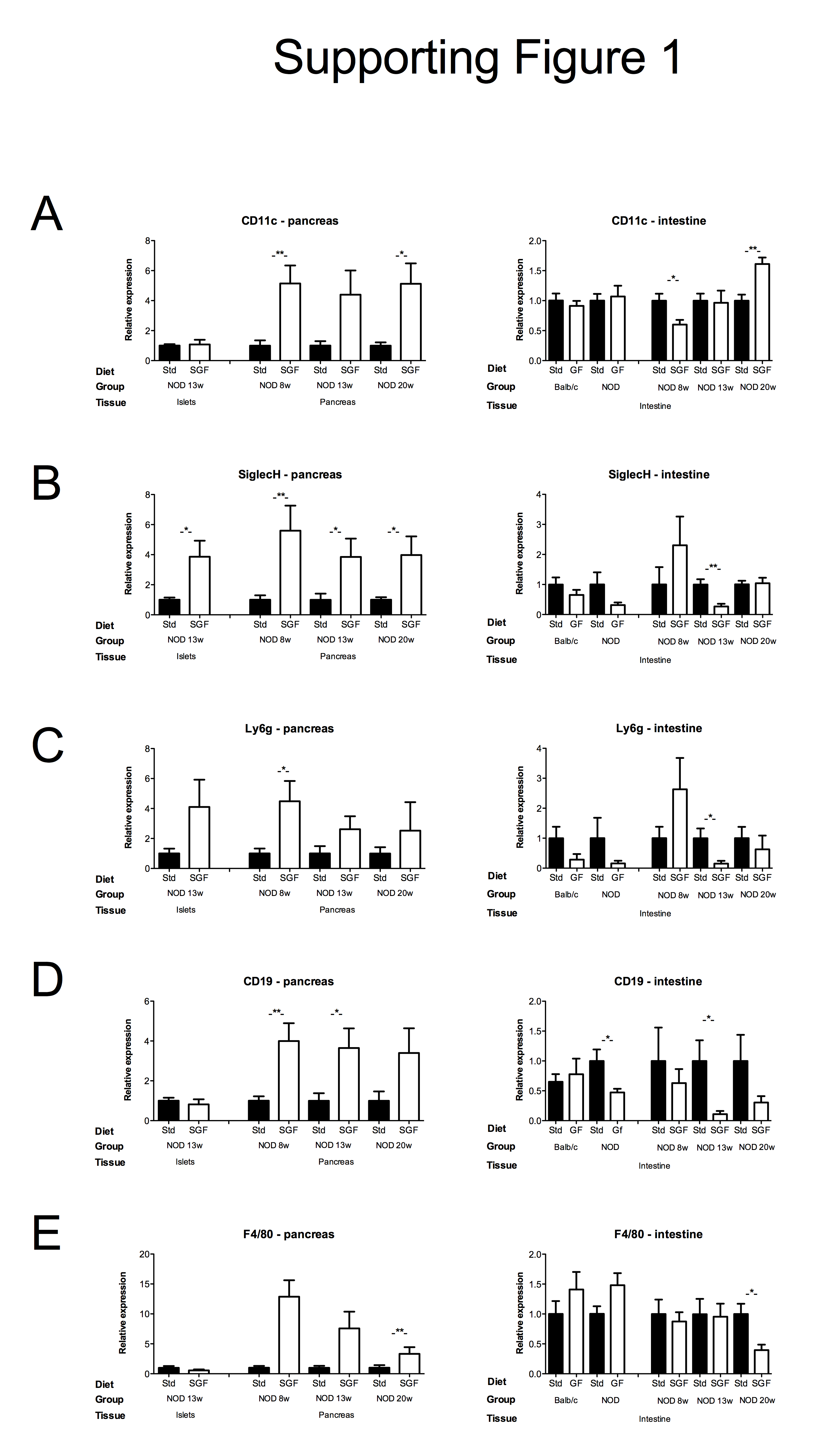

Supplement: S1 Fig — Relative mRNA expression levels of (a) CD11c, (b) SiglecH, (c) Ly6G, (d) CD19 and (e) F4/80 from 8 and 13-week old mice kept on a gluten-free (GF), strictly gluten-free (SGF), or matched control standard diet (STD) diet. Left panel: Expression levels isolated islets from SGF and STD NOD mice at 13 weeks, and expression in pancreas from SGF and STD NOD mice at 8, 13 and 20 weeks. Right panel: Expression levels in intestinal tissue from GF and STD BALB/c and NOD mice at 13 weeks, and SGF and STD NOD mice at 8, 13 and 20 weeks. Data are represented as mean values ± standard error of the mean (n = 6–12). * p < 0.05; ** p < 0.01; *** < 0.001. (TIFF) [file pone.0118618.s003.tiff]
